# Sedentary behaviour, physical activity, and renal function in older adults: isotemporal substitution modelling

**DOI:** 10.1186/s12882-020-01869-8

**Published:** 2020-06-03

**Authors:** Keisei Kosaki, Koichiro Tanahashi, Masahiro Matsui, Nobuhiko Akazawa, Yosuke Osuka, Kiyoji Tanaka, David W. Dunstan, Neville Owen, Ai Shibata, Koichiro Oka, Seiji Maeda

**Affiliations:** 1 Faculty of Sport Sciences, Waseda University, Tokorozawa, Saitama Japan; 2 Faculty of Health and Sport Sciences, University of Tsukuba, Tsukuba, Ibaraki Japan; 3 Japan Society for the Promotion of Science, Chiyoda-ku, Tokyo Japan; 4 Department of Health and Sports Sciences, Kyoto Pharmaceutical University, Kyoto, Kyoto Japan; 5 Graduate School of Comprehensive Human Sciences, University of Tsukuba, Tsukuba, Ibaraki Japan; 6 Japan Institute of Sports Sciences, Kita-ku, Tokyo Japan; 7 Research Team for Promoting Independence of the Elderly, Tokyo Metropolitan Institute of Gerontology, Itabashi-ku, Tokyo Japan; 8 Baker Heart and Diabetes Institute, Melbourne, Victoria Australia; 9 Mary MacKillop Institute for Health Research, Australian Catholic University, Melbourne, Victoria Australia; 10 Centre for Urban Transitions, Swinburne University of Technology, Hawthorn, Victoria Australia

**Keywords:** Sedentary time, Physical activity time, Estimated glomerular filtration rate, Chronic kidney disease, Isotemporal substitution modelling

## Abstract

**Background:**

Physical inactivity and sedentary behaviour (too much sitting) can contribute to renal dysfunction. However, the potential benefits of behavioural change (e.g. replacing sedentary behaviour with physical activity) on renal function are not well understood. We used isotemporal substitution to model potential impacts of behaviours on renal function by replacing time spent in one behaviour to another.

**Methods:**

In 174 older Japanese adults (age, 50–83 years; females, 76%), the time spent in sedentary behaviour, light-intensity physical activity (LPA), and moderate- to vigorous-intensity physical activity (MVPA) were assessed using an uniaxial accelerometer. Renal function was evaluated by the estimated glomerular filtration rate (eGFR) from serum creatinine and cystatin C levels.

**Results:**

In univariate analyses, eGFR was significantly, albeit weakly, correlated with time spent in sedentary behaviour (*r*_*s*_ = − 0.229), LPA (*r*_*s*_ = 0.265), and MVPA (*r*_*s*_ = 0.353). In the isotemporal substitution models, replacement of 30 min/day of sedentary behaviour with an equivalent LPA time was not significantly associated with eGFR (*β* = 2.26, *p* = 0.112); however, replacement with an equivalent time of MVPA was beneficially associated with eGFR (*β* = 5.49, *p* < 0.05).

**Conclusions:**

These cross-sectional findings suggest that sedentary behaviour (detrimentally) and physical activity (beneficially) may affect renal function and that replacing sedentary behaviour with MVPA may benefit renal health in older adults.

## Background

Chronic kidney disease (CKD), defined as persistent renal dysfunction or renal damage, is a major non-communicable disease with a high prevalence worldwide [1]. CKD in the Japanese adult population is estimated to affect approximately one in eight and has been increasing among older adults [2]. As these trends of CKD prevalence are expected to continue, especially in countries with ageing populations, including Japan [3], there is a need to identify practical countermeasures for preventing the onset and progression of CKD in older adults.

Insufficient moderate- to vigorous-intensity physical activity (MVPA) is known to be associated with the onset of renal dysfunction [4]. Subsequently, physical inactivity has been recognised recently as an important target in intervention for renal care [5]. In addition, new evidence suggests that sedentary behaviour, defined as any waking behaviour characterised by an energy expenditure ≤1.5 metabolic equivalents, such as television viewing time [6], may be another risk factor for renal dysfunction [7, 8]. Taken together, these epidemiological findings suggest the importance of behavioural approaches for renal care and the possibility that specific behavioural changes (e.g. replacing sedentary behaviour with physical activity) may contribute to preventing renal dysfunction. However, the potential impacts of replacing sedentary behaviour with physical activity on renal function are not well understood.

Isotemporal substitution modelling is a novel statistical approach that enables the estimation of associations when substituting time from one behaviour to another, while keeping the total time fixed and other behaviours fixed [9]. Recent cross-sectional studies using this approach have demonstrated that replacing time spent in sedentary behaviour with equivalent physical activities can be beneficially associated with cardiometabolic biomarkers, health-related quality of life, and physical function [10–13]. However, isotemporal substitution has not yet been used to examine the potential time-substitution effects of one behaviour for another on renal function. This is necessary to extend our current understanding of the association between sedentary behaviour, physical activity, and renal function.

Therefore, we used isotemporal substitution modelling to examine cross-sectional associations of accelerometer-derived sedentary behaviour and the different physical activities based on the intensity with renal function in older adults.

## Methods

### Participants

**Fig. 1:**
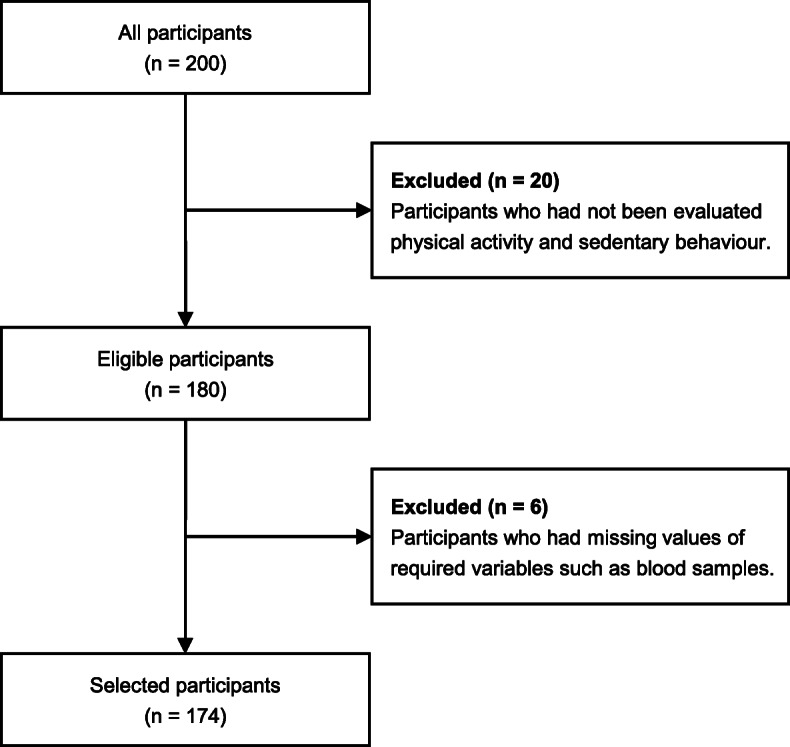
Flow diagram

The data in this cross-sectional study were collected from the baseline measurements of our previous interventional study [14] and from community-based physical examination conducted between June 2014 and August 2015 at the University of Tsukuba. All participants were recruited via local newspaper advertisements posted throughout southern Ibaraki. This study included the relatively healthy 200 participants over 50 years old and had estimated glomerular filtration rate (eGFR) measurements. In total, there were 180 eligible participants, after excluding those without objectively evaluated sedentary behaviour and physical activity or those who had insufficient accelerometer data. After excluding participants who had missing values of required variables, such as blood samples (*n* = 6), the final analyses were conducted in 174 older Japanese adults (aged 50–83 years old) (Fig. 1). This study was approved by the Ethics Committee of the University of Tsukuba (Tai 019–19) and conformed to the principles outlined in the Declaration of Helsinki. Informed consent was obtained in the form of written or opt out from all participants to use the collected data for this observational study.

### Procedures

All laboratory measurements were performed in an environmentally controlled room after adequate rest. Each participant abstained from large meals and vigorous exercise a day before the evaluation sessions. First, venous blood and spot urine samples were collected for biochemical investigations, following which blood pressure and heart rate were measured in the supine position. Subsequently, medication use by the participants, such as antihypertensive, lipid-lowering, and hypoglycaemic agents, current smoking status, and sleep time were surveyed using a self-administered questionnaire and a well-validated sleep questionnaire (the Japanese version of the Pittsburgh Sleep Quality Index). In the sleep questionnaire, the participants were instructed to record their sleep duration and sleep quality during the past month. After these procedures, accelerometers (Lifecorder, Suzuken Co., Ltd., Nagoya, Japan) were distributed to assess the participants’ daily physical activity and time spent in sedentary behaviour.

### Sedentary behaviour and physical activity (exposures)

The time spent in each kind of behaviour (i.e. sedentary behaviour, light-intensity physical activity [LPA], and MVPA) were assessed using an uniaxial accelerometer (Lifecorder, Suzuken Co., Ltd., Nagoya, Japan) which samples vertical acceleration signals in the range from 0.06 to 1.94 G at 32 Hz. The accuracy and detailed algorithm of this accelerometer have been described elsewhere [15]. The time used by the accelerometer for measuring activity, the epoch length was 1 min. Participants were instructed to wear the accelerometer at the level of the participant’s waist during waking and sleeping hours constantly for 7 consecutive days, except while bathing and swimming. A day with at least 10 h of wear time was considered valid. The accelerometer recorded the scores of intensity of activity on a scale from 0 to 9 (level 0: rest; level 0.5: micro activity; level 1–9: movement) based on acceleration signal patterns [16]. These scores were reclassified into four behavioural levels based on a previous investigation [17] as follows: sedentary or sleep (≤ 1.5 Mets: level 0–0.5), light activity (1.6–2.9 Mets: level 1–3), moderate activity (3.0–6.0 Mets: level 4–6), and vigorous activity (> 6.0 Mets: level 7–9); these were reported as the time spent in each behavioural level. The time spent in sedentary behaviour was calculated as the time of sedentary or sleep (< 1.5 Mets: level 0–0.5) minus the sleep time. In addition, moderate and vigorous physical activity time was combined to record the time spent in MVPA.

### Renal function (outcome)

Serum creatinine and cystatin C concentrations were assessed using venous blood samples collected in the morning following overnight fasting of more than 12 h. Subsequently, eGFR was calculated using the Japanese eGFR equations based on standardised serum creatinine or cystatin C levels as follows: eGFR_cr_ (mL/min/1.73 m^2^) = 194 × serum creatinine^− 1.094^ × Age^− 0.287^ (× 0.739 if female), eGFR_cys_ (mL/min/1.73 m^2^) = [104 × serum cystatin C^− 1.019^ × 0.996 ^Age^ (× 0.929 if female)] – 8 [18, 19]. To improve the estimated accuracy, the average values of eGFR_cr_ and eGFR_cys_ were used as index of renal function.

### Covariates

Brachial systolic and diastolic blood pressure and heart rate were simultaneously measured using a semi-automatic vascular testing device with electrocardiogram and oscillometric extremity cuffs (form PWV/ABI, Colin Medical technology, Komaki, Japan) with patients in the supine position. Biochemical parameters including serum or plasma concentrations of high-density lipoprotein (HDL) cholesterol, low-density lipoprotein (LDL) cholesterol, triglyceride, and blood glucose were assessed using venous blood samples collected in the morning following overnight fasting of more than 12 h. Urinary concentrations of albumin and creatinine were also assessed using spot urine samples collected the same morning as blood samples. The presence of albuminuria was defined as a urinary albumin creatinine ratio ≥ 30 mg/g.

### Statistical analysis

All statistical analyses were performed using IBM SPSS Statistics 25.0. Statistical significance was set a priori at *p* < 0.05. Data were presented as the means (SD) for normal distribution, median (interquartile range) for skewed distribution, or frequency counts [%] for categorical data, as appropriate.

Spearman’s rank correlation coefficients (*r*_*s*_) were used to examine univariate linear associations of sedentary behaviour, LPA, and MVPA with eGFR. Interactions between sedentary behaviour and physical activity were further examined using two-way analysis of covariance for assessing the adjusted effects of combined exposure to these variables on eGFR. Each participant was allocated to one of four categories based on each behaviour (four categories stratified according to each median value: higher sedentary behaviour/higher physical activity, higher sedentary behaviour/lower physical activity, lower sedentary behaviour/higher physical activity, and lower sedentary behaviour/lower physical activity). Adjusted mean eGFR values were determined for each category, adjusting for all potential covariates including total waking time, age, sex, body mass index, systolic blood pressure, heart rate, HDL cholesterol, LDL cholesterol, triglycerides, fasting blood glucose, albuminuria, medication use (antihypertensive, lipid-lowering, and hypoglycaemic agents), and current smoking status. Potential covariates were selected based on a previously recognised association in the literature or a significant univariate correlation.

Three multiple linear regression models including a single factor, partition, and isotemporal substitution model were used to examine the independent associations of the behavioural variables (sedentary behaviour, LPA, and MVPA) with the outcome variable (eGFR). For enhanced interpretability of the results, the time spent in sedentary behaviour, LPA, and MVPA were scaled to 30 min/day units [13].

The single factor model separately assessed the associations between each behavioural variable (e.g. sedentary behaviour alone) and eGFR without considering the other behavioural variables, while adjusting for total waking time and covariates (Models 1–3). An example for this model (for sedentary behaviour) is represented as follows: eGFR = (*β*_0_) sedentary behaviour + (*β*_3_) total waking time + (*β*_4_) covariates. In this case, the coefficient *β*_0_ represents the effects of increasing sedentary behaviour while keeping the total waking time constant.

The partition model simultaneously evaluated the associations between all behavioural variables and eGFR without considering the total waking time, while adjusting for covariates (Model 4). This model is represented as follows: eGFR = (*β*_0_) sedentary behaviour + (*β*_1_) LPA + (*β*_2_) MVPA + (*β*_4_) covariates. In this case, the coefficient of each behavioural variable (i.e. *β*_0_, *β*_1_, and *β*_2_) represents the effects of increasing each behaviour while keeping the other behaviour times constant. Since the total waking time is not included in this model, it merely represents the additive effects rather than the time-substitution effects of one behaviour for another.

The isotemporal substitution model estimated the substitutional associations between replacing one behavioural variable with an equal amount of another (e.g. replacement of 30 min/day of sedentary behaviour with 30 min/day of MVPA) and eGFR (Models 5–7). This estimation can be accomplished by omitting the target behavioural variable from the model and entering the total waking time and the covariates. For example, this model (with sedentary behaviour as target variable) is represented as follows: eGFR = (*β*_1_) LPA + (*β*_2_) MVPA + (*β*_3_) total waking time + (*β*_4_) covariates. In this case, the coefficients *β*_1_ and *β*_2_ represent the effects of a 30 min/day substitution of sedentary behaviour with LPA or MVPA while keeping the other behavioural variables and the total waking time constant.

## Results

The participant characteristics are shown in Table 1. The mean age was 64 ± 7 years and the majority of participants were females (76%). On average, the major part of total waking time (1056 ± 61 min/day) was spent in sedentary behaviour (970 ± 64 min/day) with 67 ± 25 min of LPA, and 19 ± 14 min of MVPA per day. Mean eGFR was 85 ± 14 mL/min/1.73 m^2^ and in a small proportion of participants (*n* = 6) it was less than 60 mL/min/1.73m^2^. Furthermore, nine participants have presented albuminuria (5%). A few participants were on medications, including antihypertensives (11%), lipid-lowering agents (6%), and/or hypoglycaemic agents (1%).

**Table 1:** Characteristics of selected participants (*n* = 174)

Variables	Total
Age, years	64 (7)
Women, n (%)	132 (76)
Height, cm	158 (7)
Weight, kg	57.1 (9.1)
Body mass index, kg/m^2^	22.9 (2.9)
Sleep time, min/day	365 (360–420)
Total waking time, min/day	1075 (1020–1080)
Sedentary behaviour, min/day	970 (64)
Light-intensity physical activity, min/day	64 (50–84)
Moderate- to vigorous-intensity physical activity, min/day	16 (9–25)
Systolic blood pressure, mmHg	122 (113–132)
Diastolic blood pressure, mmHg	75 (9)
Heart rate, bpm	61 (56–66)
High-density lipoprotein cholesterol, mg/dL	64 (14)
Low-density lipoprotein cholesterol, mg/dL	132 (29)
Triglyceride, mg/dL	82 (59–110)
Fasting blood glucose, mg/dL	91 (86–96)
eGFR, mL/min/1.73m^2^	85 (14)
Albuminuria, n [%]	9 [5]
Antihypertensive medicine, n [%]	20 [11]
Lipid-lowering medicine, n [%]	10 [6]
Hypoglycemic medicine, n [%]	1 [1]
Current smoking, n [%]	3 [2]

Data are presented as the means (SD), median (interquartile range), or frequency counts [%], as appropriate. eGFR, average value of estimated glomerular filtration rate calculated from serum creatinine or cystatin C

**Fig. 2:**
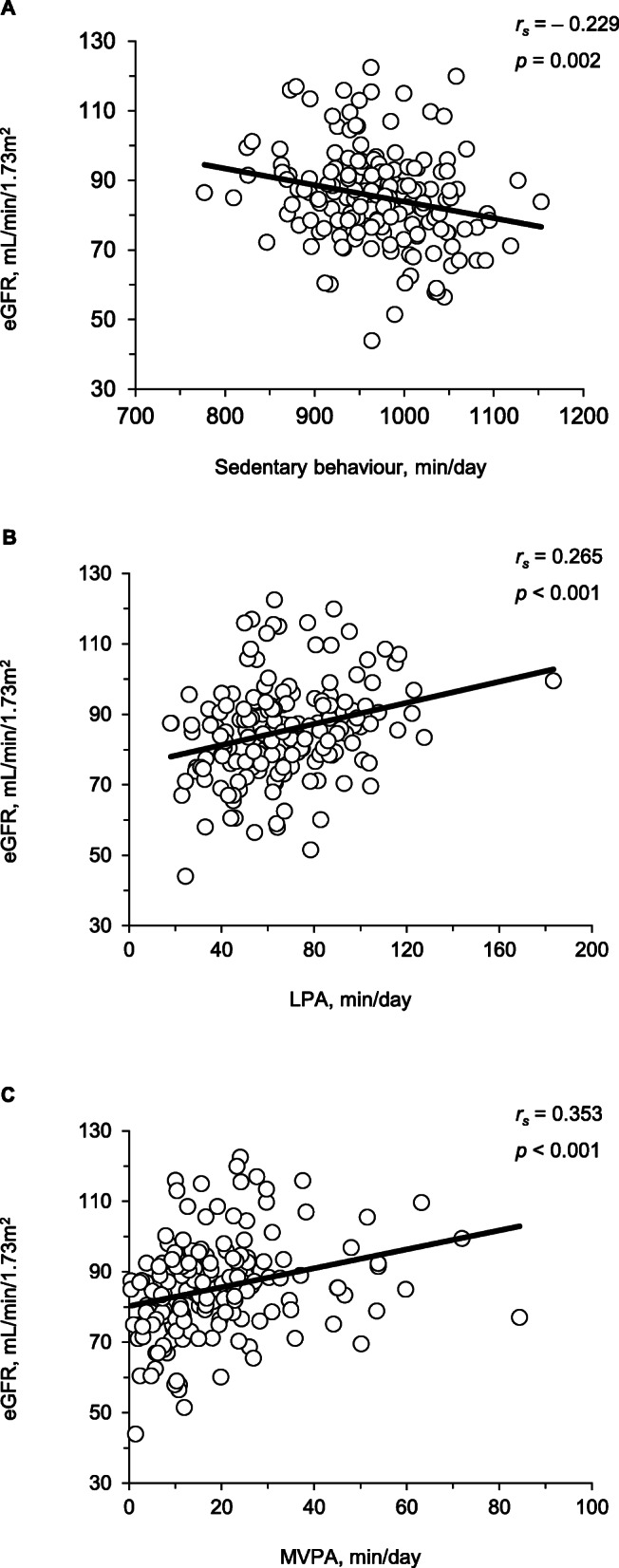
Univariate associations between sedentary behaviour (**a**), light-intensity physical activity (LPA) (**b**), moderate- to vigorous-intensity physical activity (MVPA), (**c**) and estimated glomerular filtration rate (eGFR)

The results of the univariate linear analyses are presented in Fig. 2. The time spent in sedentary behaviour was significantly and negatively, albeit weakly, correlated with eGFR (*r*_*s*_ = − 0.229, *p* = 0.002). In contrast, the time spent in LPA and MVPA were significantly and positively correlated with eGFR (*r*_*s*_ = 0.265, *p* < 0.001; *r*_*s*_ = 0.353, *p* < 0.001, respectively).

**Fig. 3:**
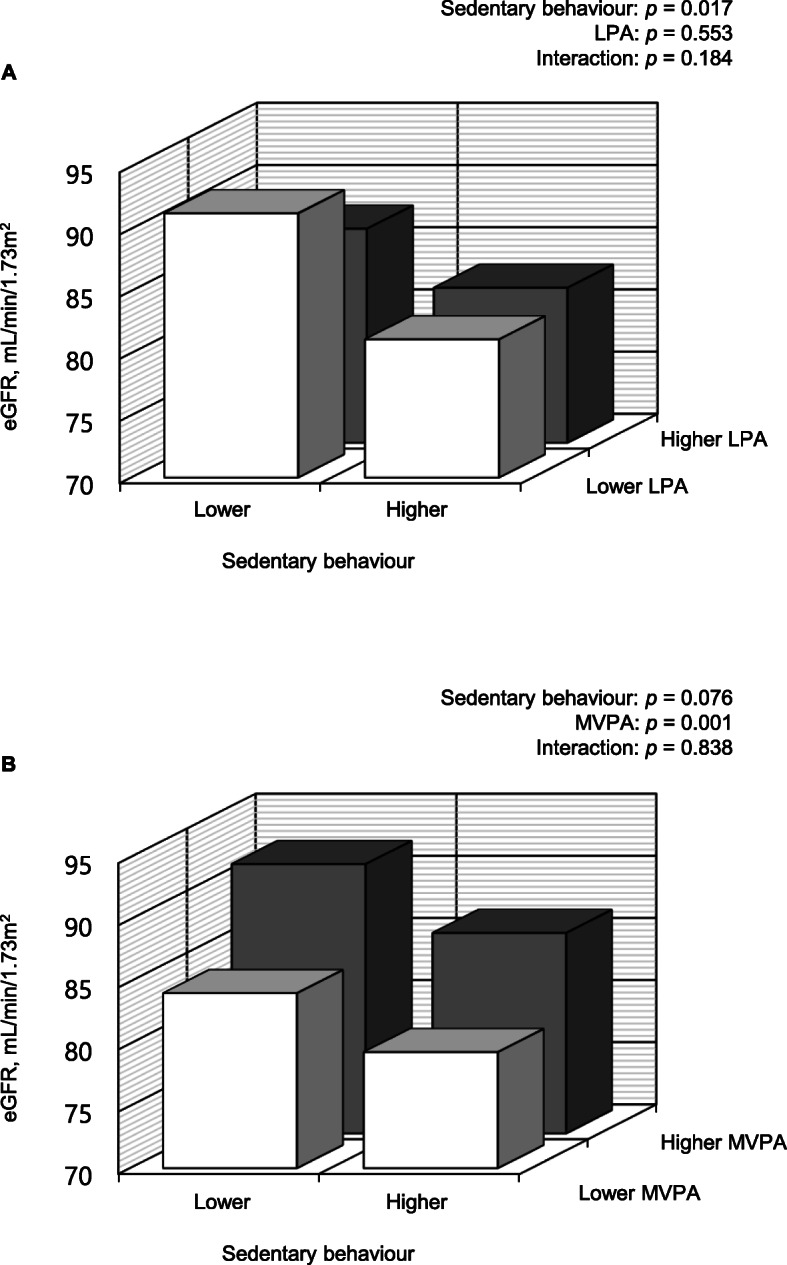
Associations of joint categories (four groups stratified according to each median value) of sedentary behaviour, light-intensity physical activity (LPA) (**a**), and moderate- to vigorous-intensity physical activity (MVPA) (**b**) with estimated glomerular filtration rate (eGFR). *p*-values were evaluated using two-way analysis of covariance after adjusting for total waking time, age, sex, body mass index, systolic blood pressure, heart rate, HDL cholesterol, LDL cholesterol, triglyceride, fasting blood glucose, albuminuria, medication use (antihypertensive, lipid-lowering, and hypoglycaemic agents), and current smoking status

Fig. 3 shows the joint associations of sedentary behaviour, LPA, and MVPA (four groups stratified according to each median value) with eGFR. Although there was no statistically significant interaction between sedentary behaviour and LPA (*p* = 0.184) or MVPA (*p* = 0.838) in their association with eGFR, the mean eGFR values were lowest in those engaged in higher sedentary behaviour and lower LPA (81.1 mL/min/1.73 m^2^; 95% CI, 76.9–85.4) or MVPA (79.3 mL/min/1.73 m^2^; 95% CI, 74.9–83.7).

The results of the three multiple linear regression models with adjustments for covariates are summarised in Table 2. The single factor models (Models 1–3) showed that sedentary behaviour was significantly and negatively associated with eGFR (*β* = − 3.31, *p* < 0.001); conversely, both LPA and MVPA were significantly and positively associated with eGFR (*β* = 3.62, *p* < 0.05; *β* = 7.06, *p* < 0.05, respectively). The partition model (Model 4) showed that only MVPA tended to be associated with eGFR (*β* = 4.68, *p* = 0.052). The isotemporal substitution models (Models 5–7) showed that replacement of 30 min/day of sedentary behaviour with an equivalent LPA time was not significantly associated with eGFR (*β* = 2.26, *p* = 0.112); however, replacement with an equivalent MVPA time was modestly but significantly associated with eGFR (*β* = 5.49, *p* < 0.05). Furthermore, replacement of 30 min/day of LPA with an equivalent MVPA time was not significantly associated with eGFR (*β* = 3.23, *p* = 0.317).

**Table 2:** Single factor, partition, and isotemporal substitution models for renal function represented by eGFR

Models		Sedentary behaviour	LPA	MVPA	R-Square
		*β* (95% CI)	*β* (95% CI)	*β* (95% CI)	
Single factor model^a,b^	1	– 3.31 (− 5.17,– 1.45)^**^			0.262^**^
	2		3.62 (1.04,6.19)^*^		0.242^**^
	3			7.06 (2.79,11.34)^*^	0.255^**^
Partition model^a^	4	– 0.81 (− 1.77,0.16)	1.45 (− 1.33,4.24)	4.68 (− 0.04,9.40)	0.267^**^
Isotemporal substitution model^a,b^	5	Dropped	2.26 (− 0.53,5.05)	5.49 (0.81,10.17)^*^	0.267^**^
	6	– 2.26 (− 5.05,0.53)	Dropped	3.23 (− 3.13,9.60)	0.267^**^
	7	– 5.49 (− 10.17,– 0.81)^*^	– 3.23 (− 9.60,3.13)	Dropped	0.267^**^

^a^All models adjusted for age, sex, body mass index, systolic blood pressure, heart rate, high-density lipoprotein cholesterol, low-density lipoprotein cholesterol, triglyceride, fasting blood glucose, albuminuria, medication use (antihypertensive, lipid-lowering, and hypoglycaemic agents), and current smoking. ^b^single factor and isotemporal substitution models were also adjusted for total waking time. Regression coefficients (*β*) correspond to a 30 min/day of each behaviour. ^**^p < 0.001, ^*^p < 0.05. LPA, light-intensity physical activity; MVPA, moderate- to vigorous-intensity physical activity

## Discussion

In this cross-sectional study of older adults, we examined the associations of accelerometer-derived sedentary behaviour and different intensities of physical activity with renal function and the potential renal impacts of replacing sedentary behaviour with different intensities of physical activity. Time spent in sedentary behaviour, LPA, and MVPA were significantly associated with eGFR. Furthermore, isotemporal substitution modelling showed that replacement of 30 min/day of sedentary behaviour with an equivalent MVPA time was significantly associated with improved eGFR. These findings suggest that both sedentary behaviour (detrimentally) and physical activity (beneficially) may be contributing factors affecting renal function and replacing sedentary behaviour with physical activity (MVPA) may benefit renal health in older adults.

Several previous studies have reported the associations of sedentary behaviour and physical activity with renal function [7, 8, 20–26]. However, only a few studies have used accelerometer-derived measures of sedentary behaviour and physical activity [20, 21, 24], and most studies have relied on self-reported measures derived from questionnaires, which may be limited in accurately quantifying the behavioural factors. In addition, no study has examined the potential impacts of replacing sedentary behaviour with physical activity on renal function using isotemporal substitution modelling. To our knowledge, this is the first study to examine the associations of accelerometer-derived sedentary behaviour and physical activity with renal function using isotemporal substitution modelling in Japanese adults who have been reported to exhibit the highest sitting time (medians ≥360 min/day) [27].

This study also showed that the mean values of eGFR were lowest in those with higher sedentary behaviour and lower physical activity (LPA and MVPA). However, there was no statistically significant interaction between sedentary behaviour and physical activity with respect to the association with eGFR. This is because the influence of physical activity, especially MVPA on eGFR, might be apparently stronger than that of sedentary behaviour. Univariate analyses also showed that the associations between MVPA and eGFR (*r*_*s*_ = 0.353) were stronger than those of sedentary behaviour (*r*_*s*_ = − 0.229). Therefore, the association of a combination of higher sedentary behaviour and lower physical activity is likely to be strongest with poorer renal function; however, MVPA emerged as potentially having a more significant influence on renal function than sedentary behaviour.

Beneficial effects of regularly performed physical activity (i.e. exercise training) on several medical conditions have been demonstrated in the general population and in patients with CKD. The Kidney Disease: Improving Global Outcomes (KDIGO) guidelines mainly intend to maintain and improve cardiovascular health and tolerance and recommend that patients with CKD should undertake moderate physical activity for at least 30 min five times per week, in line with recommendations for the general population [28]. Furthermore, recent meta-analyses have also shown the possibility that exercise training slightly improves eGFR in patients with CKD [29, 30]. However, the beneficial effects of exercise training on renal function are not likely to be conclusive and remain controversial due to a limited number of studies in both meta-analyses. We estimated the potential impacts of specific behavioural changes on renal function using isotemporal substitution modelling and revealed that replacement of 30 min/day of sedentary behaviour with an equivalent physical activity (MVPA) was significantly associated with improved eGFR (+ 5.49 mL/min/1.73 m^2^; 95% CI, 0.81–10.17). This finding supports and expands on the results of several previous interventional studies and the KDIGO recommendation of physical activity.

However, several noteworthy limitations need to be discussed. First, this study was a cross-sectional investigation with a relatively small sample size, which strongly limits our ability to assess the precise causal links of sedentary behaviour, physical activity, and renal function. We cannot deny the possibility that renal function may be a contributing factor in levels of sedentary behaviour and physical activity of individuals in daily life. Future longitudinal studies with a larger sample size examining the impact of a sedentary lifestyle on decline in renal function are needed to provide a more definitive interpretation of the present findings. Second, the potential renal impacts examined using the isotemporal substitution models were only estimations, and the actual impacts of specific behavioural changes on renal function remain unknown. Furthermore, the results of isotemporal substitution modelling may support the already existing hypothesis that physical activity may attenuate the loss of renal function (i.e. eGFR); however, it does not lead to the generation of a new hypothesis. Third, as the participants in this study were relatively healthy, with a substantially normal eGFR and without albuminuria, our findings may not apply to a population with persistent renal dysfunction or renal damage (i.e. patients with CKD). Furthermore, a generalization of our results may also be difficult considering our study was a single-centre study and the selective population (i.e. older Japanese adults) we recruited. Fourth, although the participants in this study were relatively healthy older adults, potential comorbidities such as frailty status can be confounded by association of sedentary behaviour, physical activity, and renal function. However, adjusting for these potential comorbidities in this study was not feasible because the relevant objective data were not available for all participants. Finally, the reasons for improved renal function on replacing sedentary behaviour with physical activity remain equivocal. Considering the findings of previous basic research [31], several mediating factors, including anti-oxidant defence and anti-inflammatory environment, may be involved in the mechanism underlying the renal benefits of specific behavioural changes. Further interventional studies, such as randomised controlled trials, examining the effects of moderate- to vigorous-intensity exercise on renal health are needed to address these limitations. The strengths of this study are the use of accelerometer to objectively assess each behaviour and the clinically interpretable results obtained from isotemporal substitution modelling.

## Conclusion

Sedentary behaviour (detrimentally) and physical activity (LPA and MVPA) (beneficially) were significantly associated with renal function represented by eGFR in older Japanese population. Findings from isotemporal substitution modelling suggest that replacement of 30 min/day of sedentary behaviour with an equivalent MVPA time may contribute to improved renal function in older adults. These findings provide significant preliminary evidence on the potential renal benefits of decreasing sedentary time and increasing physical activity time and include important several public health implications.

## Data Availability

The datasets generated and/or analysed during the current study are not publicly available due to the ongoing nature of this study but are available from the corresponding author on reasonable request.

## Abbreviations

CKD: Chronic kidney disease
eGFR: Estimated glomerular filtration rate
HDL: High-density lipoprotein
KDIGO: Kidney Disease: Improving Global Outcomes
LDL: Low-density lipoprotein
LPA: Light-intensity physical activity
MVPA: Moderate- to vigorous-intensity physical activity

## References

[1] Xie Y, Bowe B, Mokdad AH, Xian H, Yan Y, Li T, Maddukuri G, Tsai CY, Floyd T, Al-Aly Z (2018). Analysis of the global burden of disease study highlights the global, regional, and national trends of chronic kidney disease epidemiology from 1990 to 2016. Kidney Int.

[2] Imai E, Horio M, Iseki K, Yamagata K, Watanabe T, Hara S, Ura N, Kiyohara Y, Hirakata H, Moriyama T (2007). Prevalence of chronic kidney disease (CKD) in the Japanese general population predicted by the MDRD equation modified by a Japanese coefficient. Clin Exp Nephrol.

[3] Jha V, Garcia-Garcia G, Iseki K, Li Z, Naicker S, Plattner B, Saran R, Wang A, Yang C (2013). Chronic kidney disease: global dimension and perspectives. Lancet.

[4] Hallan S, de Mutsert R, Carlsen S, Dekker FW, Aasarod K, Holmen J (2006). Obesity, smoking, and physical inactivity as risk factors for CKD: are men more vulnerable?. Am J Kidney Dis.

[5] Zelle DM, Klaassen G, van Adrichem E, Bakker SJ, Corpeleijn E, Navis G (2017). Physical inactivity: a risk factor and target for intervention in renal care. Nat Rev Nephrol.

[6] Tremblay MS, Aubert S, Barnes JD, Saunders TJ, Carson V, Latimer-Cheung AE, Chastin SFM, Altenburg TM, Chinapaw MJM, Participants STCP (2017). Sedentary behaviour research network (SBRN) - terminology consensus project process and outcome. Int J Behav Nutr Phys Act.

[7] Hawkins M, Newman AB, Madero M, Patel KV, Shlipak MG, Cooper J, Johansen KL, Navaneethan SD, Shorr RI, Simonsick EM (2015). TV watching, but not physical activity, is associated with change in kidney function in older adults. J Phys Act Health.

[8] Lynch BM, White SL, Owen N, Healy GN, Chadban SJ, Atkins RC, Dunstan DW (2010). Television viewing time and risk of chronic kidney disease in adults: the AusDiab study. Ann Behav Med.

[9] Mekary RA, Willett WC, Hu FB, Ding EL (2009). Isotemporal substitution paradigm for physical activity epidemiology and weight change. Am J Epidemiol.

[10] Yasunaga A, Shibata A, Ishii K, Inoue S, Sugiyama T, Owen N, Oka K (2018). Replacing sedentary time with physical activity: effects on health-related quality of life in older Japanese adults. Health Qual Life Outcomes.

[11] Yasunaga A, Shibata A, Ishii K, Koohsari MJ, Inoue S, Sugiyama T, Owen N, Oka K (2017). Associations of sedentary behaviour and physical activity with older adults' physical function: an isotemporal substitution approach. BMC Geriatr.

[12] Hamer M, Stamatakis E, Steptoe A (2014). Effects of substituting sedentary time with physical activity on metabolic risk. Med Sci Sports Exerc.

[13] Buman MP, Winkler EA, Kurka JM, Hekler EB, Baldwin CM, Owen N, Ainsworth BE, Healy GN, Gardiner PA (2014). Reallocating time to sleep, sedentary behaviours, or active behaviours: associations with cardiovascular disease risk biomarkers, NHANES 2005-2006. Am J Epidemiol.

[14] Osuka Y, Fujita S, Kitano N, Kosaki K, Seol J, Sawano Y, Shi H, Fujii Y, Maeda S, Okura T (2017). Effects of aerobic and resistance training combined with fortified Milk on muscle mass, muscle strength, and physical performance in older adults: a randomized controlled trial. J Nutr Health Aging.

[15] Kumahara H, Schutz Y, Ayabe M, Yoshioka M, Yoshitake Y, Shindo M, Ishii K, Tanaka H (2004). The use of uniaxial accelerometry for the assessment of physical-activity-related energy expenditure: a validation study against whole-body indirect calorimetry. Br J Nutr.

[16] Hikihara Y, Tanaka S, Ohkawara K, Ishikawa-Takata K, Tabata I (2012). Validation and comparison of 3 accelerometers for measuring physical activity intensity during nonlocomotive activities and locomotive movements. J Phys Act Health.

[17] Ishii K, Shibata A, Adachi M, Mano Y, Oka K (2017). Objectively measured sedentary behaviour, obesity, and psychological well-being: a cross-sectional study of Japanese schoolchildren. J Phys Act Health.

[18] Horio M, Imai E, Yasuda Y, Watanabe T, Matsuo S (2013). Collaborators developing the Japanese equation for estimated GFR: GFR estimation using standardized serum cystatin C in Japan. Am J Kidney Dis.

[19] Matsuo S, Imai E, Horio M, Yasuda Y, Tomita K, Nitta K, Yamagata K, Tomino Y, Yokoyama H, Hishida A (2009). Revised equations for estimated GFR from serum creatinine in Japan. Am J Kidney Dis.

[20] Martens RJH, van der Berg JD, Stehouwer CDA, Henry RMA, Bosma H, Dagnelie PC, van Dongen M, Eussen S, Schram MT, Sep SJS (2018). Amount and pattern of physical activity and sedentary behaviour are associated with kidney function and kidney damage: the Maastricht study. PLoS One.

[21] Glavinovic T, Ferguson T, Komenda P, Rigatto C, Duhamel TA, Tangri N, Bohm C (2018). CKD and sedentary time: results from the Canadian health measures survey. Am J Kidney Dis.

[22] Robinson-Cohen C, Littman AJ, Duncan GE, Weiss NS, Sachs MC, Ruzinski J, Kundzins J, Rock D, de Boer IH, Ikizler TA (2014). Physical activity and change in estimated GFR among persons with CKD. J Am Soc Nephrol.

[23] Bharakhada N, Yates T, Davies MJ, Wilmot EG, Edwardson C, Henson J, Webb D, Khunti K (2012). Association of sitting time and physical activity with CKD: a cross-sectional study in family practices. Am J Kidney Dis.

[24] Hawkins MS, Sevick MA, Richardson CR, Fried LF, Arena VC, Kriska AM (2011). Association between physical activity and kidney function: National Health and nutrition examination survey. Med Sci Sports Exerc.

[25] Robinson-Cohen C, Katz R, Mozaffarian D, Dalrymple LS, de Boer I, Sarnak M, Shlipak M, Siscovick D, Kestenbaum B (2009). Physical activity and rapid decline in kidney function among older adults. Arch Intern Med.

[26] Finkelstein J, Joshi A, Hise MK (2006). Association of physical activity and renal function in subjects with and without metabolic syndrome: a review of the third National Health and nutrition examination survey (NHANES III). Am J Kidney Dis.

[27] Bauman A, Ainsworth BE, Sallis JF, Hagstromer M, Craig CL, Bull FC, Pratt M, Venugopal K, Chau J, Sjostrom M (2011). The descriptive epidemiology of sitting. A 20-country comparison using the international physical activity questionnaire (IPAQ). Am J Prev Med.

[28] Levin A, Stevens PE (2014). Summary of KDIGO 2012 CKD guideline: behind the scenes, need for guidance, and a framework for moving forward. Kidney Int.

[29] Yamagata K, Hoshino J, Sugiyama H, Hanafusa N, Shibagaki Y, Komatsu Y, Konta T, Fujii N, Kanda E, Sofue T, et al. Clinical practice guideline for renal rehabilitation: systematic reviews and recommendations of exercise therapies in patients with kidney diseases. Renal Replacement Therapy. 2019;5(1).

[30] Vanden Wyngaert K, Van Craenenbroeck AH, Van Biesen W, Dhondt A, Tanghe A, Van Ginckel A, Celie B, Calders P (2018). The effects of aerobic exercise on eGFR, blood pressure and VO2peak in patients with chronic kidney disease stages 3-4: a systematic review and meta-analysis. PLoS One.

[31] Asghar M, George L, Lokhandwala MF (2007). Exercise decreases oxidative stress and inflammation and restores renal dopamine D1 receptor function in old rats. Am J Physiol Ren Physiol.

